# Supplementary material for: Comparison of the internal fit of metal crowns fabricated by traditional casting, computer numerical control milling, and three-dimensional printing
Source: PLoS One. 2021 Sep 16;16(9):e0257158. doi: 10.1371/journal.pone.0257158 (PMC8445422; doi:10.1371/journal.pone.0257158)
Supplement: Attachment — Submitted filename: Response_to_Reviewers.doc [file pone.0257158.s002.doc]

**Response to Reviewers**

1) Please add a summary of technology used to mill and print metal crowns in the introduction, perhaps in the third paragraph.
**Response:** In simple terms, the process can be described as follows: the dentist creates a digital dataset on the computer (computer-aided design, CAD) and then designs a 3D object; the data of the 3D object are then transferred to the milling machine or a 3D printer, which creates a physical object from these data.

2) Please add explicit hypothesis and what you have expected in the last part of the introduction.
**Response:** The null hypothesis is that the internal fit of digitally fabricated crowns is better than those of traditionally fabricated crowns.

3) The Discussion needs to explain the limitations of the study and how these limitations may be addressed in future experiments. This should be in the last part of the Discussion. Errors of the lost wax technique, scanning errors, printing errors, and measurement errors should also be discussed.
**Response:** The limitations of this study include the *in vitro* setting, which might not reflect real conditions in patients’ mouths and daily clinical practice. In addition, even though the CAM equipment of the CAD/CAM system, which included the milling machine and 3D printer, has extremely outstanding surface illumination and high precision, more specimens and various milling machines and printers were not used. Therefore, there is a need to secure a greater number of specimens and additional equipment in future studies, and the use of human teeth would be ideal for simulating a clinical procedure.

4) Please discuss clinical relevances and how the experiment may or may not applicable to clinic patients.
**Response:** In the traditional casting method, the molten alloy used for crown restoration shrinks upon solidification, and cobalt-chromium alloys shrink by as much as 2%. If the mold is not made correspondingly larger than the original wax pattern, the resultant cast will be much smaller. Therefore, for casting crowns, it is necessary to compensate for the solidification-related shrinkage of the alloy used by expanding the mold size to at least equal the shrinkage size. One of the mechanisms to produce mold expansion is wax pattern expansion; therefore, proper control by technicians may also influence the crown fit result.

Over the years, the CAD/CAM fabrication method has gained popularity over the conventional method in dentistry, and dentists are moving ahead with the trend by taking advantage of the opportunities provided by the CAD/CAM technology to equip their clinics or laboratories with the latest materials that will make work easier and more efficient. One of the benefits of dental CAD/CAM technology is that it is time-saving, enabling faster dental crown production. It reduces the time to produce crowns to 2-5 days instead of 2-3 weeks required with the conventional procedure. With the CAD/CAM technology in dentistry, the outcome of restorations can be predicted. Devoid of traditional impressions requiring several processes, intraoral-extraoral scanning gives patients a more convenient dental restoration experience in the clinic.
